# Molecular Hydrogen Therapy Ameliorates Organ Damage Induced by Sepsis

**DOI:** 10.1155/2016/5806057

**Published:** 2016-06-20

**Authors:** Yijun Zheng, Duming Zhu

**Affiliations:** Department of Critical Care Medicine, Zhongshan Hospital, Fudan University, Shanghai 200032, China

## Abstract

Since it was proposed in 2007, molecular hydrogen therapy has been widely concerned and researched. Many animal experiments were carried out in a variety of disease fields, such as cerebral infarction, ischemia reperfusion injury, Parkinson syndrome, type 2 diabetes mellitus, metabolic syndrome, chronic kidney disease, radiation injury, chronic hepatitis, rheumatoid arthritis, stress ulcer, acute sports injuries, mitochondrial and inflammatory disease, and acute erythema skin disease and other pathological processes or diseases. Molecular hydrogen therapy is pointed out as there is protective effect for sepsis patients, too. The impact of molecular hydrogen therapy against sepsis is shown from the aspects of basic vital signs, organ functions (brain, lung, liver, kidney, small intestine, etc.), survival rate, and so forth. Molecular hydrogen therapy is able to significantly reduce the release of inflammatory factors and oxidative stress injury. Thereby it can reduce damage of various organ functions from sepsis and improve survival rate. Molecular hydrogen therapy is a prospective method against sepsis.

## 1. Introduction

Sepsis is a systematic inflammatory response to infection. It is one of the most serious diseases in ICU, which is a worldwide challenge. Although comprehensive therapy has been developed for it, sepsis is still associated with high morbidity and mortality and costs a lot for hospitalization. In the United States, severe sepsis affects 750,000 people per year, which costs $16.7 billion annually and increases in its incidence over time of 8.7% [1, 2].

Sepsis leads to abnormal blood pressure, heart rate, and PaO_2_. It also influences different organs and even leads to multiple organ dysfunction syndromes (MODS). The main clinical characteristics of brain include delirium, coma, disorientation, the slowing of mental processes, and cognitive dysfunction. Sepsis leads to acute lung injury and acute respiratory distress syndrome (ARDS), whose mortality rate is as high as 30% to 50% in critically ill patients. In liver, disruption of protein synthetic function manifests as progressive disruption of blood clotting and disruption of metabolic functions leads to impaired bilirubin metabolism. The incidence of acute kidney injury is nearly 65% in critically ill patients and is able to aggravate the condition of patients with septic shock. Sepsis also decreases the blood flow of the gastrointestinal tract, which might induce severe ischemia, hypoxia, and reperfusion injury.

Recent research suggests that molecular hydrogen works as a therapeutic antioxidant activity by selectively reducing hydroxyl radicals and protects against organ damage effectively. In 2007, inhalation of hydrogen gas was found to suppress brain injury by buffering the effects of oxidative stress in an acute focal ischemia and reperfusion rat mode [3]. In 2008, hydrogen therapy was found to inhibit the inflammatory reaction in the rat model of small intestinal transplantation. Contemporarily, hydrogen therapy was proved to protect against acute pancreatitis [4]. In 2010, hydrogen's protective effects on sepsis and sepsis-associated organ damage were found, which mainly relied on its antioxidative property [5].

There are 3 main methods of molecular hydrogen therapy: inhalation of hydrogen (H_2_), oral intake of hydrogen-rich water (HRS), and injection of hydrogen-saturated saline (HRS). Molecular hydrogen therapy also can be combined with other therapy such as resuscitation and oxygen therapy.

## 2. Oxidative Stress in Sepsis

Autoimmune injury occurs in sepsis, and the pathogenesis is very complicated, in which oxidative stress plays an important role.

The immunocyte is activated and the respiratory burst creates amount of reactive oxygen species (ROS). Oxidative stress induced by ROS can change the permeability of epithelial cells by destroying the cell membrane. The imbalance of antioxidant defense systems against oxidative stress also can damage the epithelial cells [6].

## 3. Mechanism of Molecular Hydrogen

Molecular hydrogen is a scavenger of the hydroxyl radical. H_2_ can selectively reduce ROS in vitro; it will react with only the strongest oxidants, which means the use of H_2_ is mild enough having no serious side effects [3].

**Figure 1:**
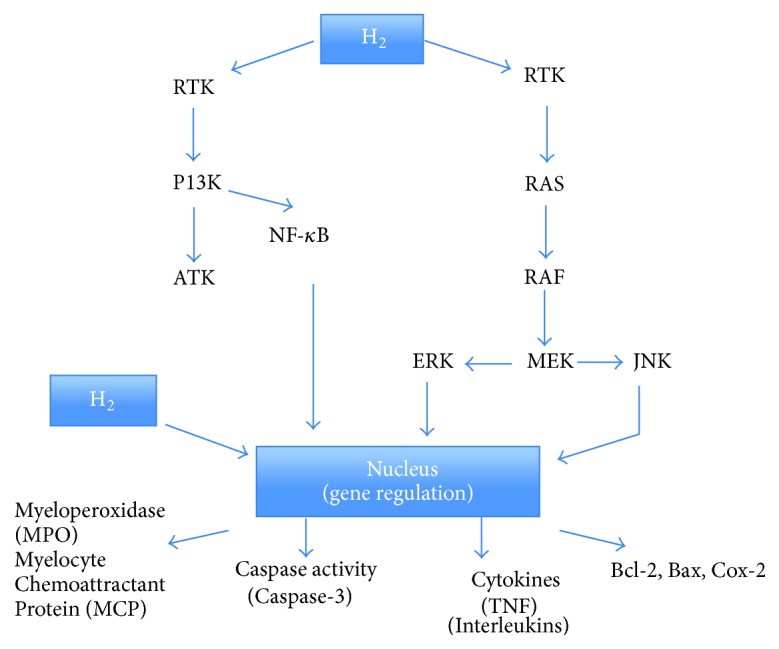
Possible mechanisms of molecular hydrogen. Possible pathways for molecular hydrogen. It has been proposed that molecular hydrogen has the capabilities to affect the pathways mentioned and to directly or indirectly assist in the gene regulation or protein expression of the following: MPO, MCP, Caspase-3, Caspase-12, TNF, interleukins, Bcl-2, Bax, and Cox-2.

Molecular hydrogen can suppress the release of cell adhesion molecules, as well as proinflammatory cytokines. H_2_ could elevate anti-inflammatory cytokine levels. H_2_ enhanced HO-1 expression and activity, which suggest that H_2_ could suppress excessive inflammatory responses and endothelial injury via an Nrf2 (nuclear factor erythroid 2 p45 related factor 2)/HO-1 pathway [14]. In addition, it has been proposed that molecular hydrogen has the capabilities to affect several pathways and assist in the gene regulation or protein expression of MPO (myeloperoxidase), MCP, Caspase-3, Caspase-12, TNF (tumor necrosis factor), interleukins, Bcl-2, Bax, and Cox-2 (as shown in Figure 1 [15]).

## 4. The Impact of Molecular Hydrogen on General Condition

In animal experiments, sepsis alters general condition of mice, such as mean artery pressure (MAP) decreasing and PaO_2_ declining.

In Liu et al.'s study, MAP decreased in 20 minutes after LPS (Lipopolysaccharide) injection. There was no significant difference in resuscitation group and resuscitation+H_2_ group, while the fluid volume and usage of norepinephrine were less used in resuscitation+H_2_ group [6]. In another study, resuscitation group needs more fluid and norepinephrine than H_2_ group although these two groups get to similar MAP [13].

Many studies showed that sepsis makes PaO_2_ and PaO_2_/FiO_2_ decline, and molecular hydrogen therapy can alleviate this change. Xie et al. stated that PaO_2_/FiO_2_ ratio declined significantly in cecal ligation and puncture (CLP) group. Inhalation of H_2_ can remit the change [10]. PaO_2_ declined in Liu et al.'s study of septic mice. Resuscitation improved PaO_2_ to 62.34 ± 2.46 mmHg (*p* < 0.05), but resuscitation+H_2_ inhalation showed more effectivity by improving PaO_2_ to 88.98 ± 3.17 mmHg [6]. When molecular hydrogen therapy is used alone, it is also valid. Li et al. stated that HRS increased PaO_2_ of 59 ± 6 mmHg to 67 ± 8 mmHg (*p* < 0.05) in CLP mice [11]. Xie et al. showed PaO_2_/FiO_2_ significantly decreased in LPS-challenged mice, which improved by H_2_ inhalation [12].

Generally, sepsis decreased MAP, PaO_2_, and PaO_2_/FiO_2_. Traditional resuscitation can alleviate these changes. It works better while combined with molecular hydrogen therapy. Molecular hydrogen therapy makes it possible for using less fluid and the vasoactive agent to reach the target MAP level. Molecular hydrogen therapy also significantly improves PaO_2_ and PaO_2_/FiO_2_ in sepsis.

## 5. The Impact of Molecular Hydrogen on Different Organs

The impact of molecular hydrogen on changes of the biochemistry indicator level in different organs was summarized in Table 1.

### 5.1. Brain

Brain is one of the organs to be affected during early sepsis. It is strongly associated with higher mortality and lower quality of life.

Morphologic changes can be detected by pathologic examination. Brain sections were stained with H&E (hematoxylin-eosin staining). In normal condition, the hippocampal CA1 region shows tightly arranged nerve cell bodies with clear structures; cytoplasm in cells is plentiful. However, animal experiments found that most neurons in CLP-challenged mice were shrunken and stained dark; the intracellular space was enlarged. With HRS injection, the cells with eumorphism were significantly preserved. Total normal cell count in sham group was 295.50 ± 12.91, while the number in CLP group was significantly decreased. Compared with CLP group, the numbers of normal cells were much higher in HRS treatment group. This data revealed the dose-response relationship of HRS treatment [7]. Inhalation of H_2_ stated similar consequence with HRS treatment. In Liu et al.'s study, the pyramidal neurons in hippocampal CA1 region were arranged in disorder in CLP group, containing the dissolved Nissl bodies. This disorder was slighter in H_2_ inhalation group. A mass of apoptotic cells in hippocampal CA1 region was found in CLP group, and there were fewer apoptotic cells found in H_2_ inhalation group [8]. Zhou et al.'s study also confirmed the result [7].

Both immunohistochemical staining of cleaved Caspase-3 and western blot of cleaved Caspase-3 expression in hippocampus indicate a great increase in CLP group. With HRS therapy Caspase-3 was dramatically reduced after the CLP event. The number of cleaved Caspase-3-positive cells was 223.62 ± 25.71 in CLP group, which was significantly greater than the sham group. Nevertheless, the numbers in 2.5 mg/kg and 10 mg/kg HRS treatment group were 142.26 ± 9.89 and 84.13 ± 12.48, respectively. Significant differences were found in those groups [7].

H_2_ treatment can attenuate blood-brain barrier disruption. Evans blue (EB) is a dye binding to serum albumin, which can seldom go through the Blood Brain Barrier (BBB). But in CLP group, obvious rise of EB quantification was observed compared with sham group; H_2_ treatment group showed less EB quantification compared with CLP group (*p* < 0.001). H_2_ treatment can also reduce brain water content. The brain water content was 74.85 ± 0.75 in sham group, which increased to 78.34 ± 0.82 (%) in CLP group (*p* < 0.001) and to 76.57 ± 0.87 (%) in H_2_ treatment group [8].

HRS treatment and H_2_ treatment prevented the abnormal changes of oxidation and antioxidation. Septic mice had lower levels of SOD and higher levels of ROS and MDA (Malondialdehyde); both HRS treatment and H_2_ treatment can prevent those changes. There was also a dose-response relationship showed in studies [7]. Moreover, activities of antioxidant enzymes (SOD and CAT) in both serum and hippocampus were significantly diminished in CLP group. On the contrary, the levels of oxidative products (MDA and 8-iso-PGF2*α*) were markedly increased. H_2_ treatment could upregulate the expression of Nrf2, which is an important transcription factor of antioxidant stress to lighten those abnormal changes. This finding may explain the antioxidant effects of molecular hydrogen therapy [8, 9].

H_2_ inhalation can significantly decrease the levels of proinflammatory cytokines (TNF-*α*, IL-1*β*, and HMGB1) and increase the level of anti-inflammatory cytokines (IL-10) (*p* < 0.001), in both serum and hippocampus [8].

Researchers measured the cognitive function of CLP-challenged mice with several methods. In Y-maze test and Fear conditioning test, H_2_ group showed higher cognitive function at days 3 to 14 after CLP operation [8]. The Morris water maze test results in cognitive impairment in CLP group; HRS injection could alleviate cognitive impairment. When the dosage of HRS boosts into 10 mL/kg, no significant difference was found between sham group and HRS treatment group. Interestingly, cognitive dysfunction recovered 10 days after the CLP event. It indicated that forced exercise may influence learning and memory.

In conclusion, sepsis can destroy the structure of brain especially hippocampus CA1 region through stimulating oxidative stress reaction and inflammatory response, which lead to impairment of cognition. However, molecular hydrogen therapy was proved to attenuate the disruption.

### 5.2. Lung

Acute lung injury/acute respiratory distress syndrome (ALI/ARDS) are common syndromes in sepsis. When ALI occurs, the oxygenation index, lung MPO activity, lung W/D weight ratio, BAL (bronchoalveolar lavage) and total protein, lung's histology, antioxidant enzymatic activity, and inflammatory cytokines are all different from normal conditions.

The normal lung structure has no hyperemia, neutrophil infiltration. But in sepsis, there can be found disordered alveolar structures, collapse of alveoli, incomplete alveolar wall, severe neutrophil infiltration, alveolar capillary congestion, and thickened alveolar wall by edema. Resuscitation only can reduce neutrophil accumulation and the alveolar-capillary exudate but cannot alleviate alveolar edema. When combined with H_2_ inhalation, the therapy significantly decreased in alveolar damage and alveolar edema as well [6]. In addition, HRS administration individually could also decrease infiltration of neutrophils, interstitial edema, and atelectasis [11]. H_2_ inhalation is confirmed to be effective as well to attenuate sepsis-induced lung injury in mice. 2% H_2_ treatment resulted in reduction of inflammatory cells infiltration and improvement in lung structure [5, 12].

Moreover, effects of H_2_ treatment on pulmonary cell apoptosis were investigated. Numerous lung cells were positive for TUNEL (TdT-mediated dUTP Nick-End Labeling) staining which identified apoptotic cells in LPS group. In the samples of H_2_ treatment group, a few of positive cells were observed. Caspase-3 detection showed the same tendency in those groups. Those data revealed LPS-induced septic stimulated pulmonary cell apoptosis and H_2_ therapy would prevent this process [12].

Lung W/D weight ratio is an indicator of the magnitude of pulmonary edema. Septic lung showed higher W/D ratios in all studies. Resuscitation+H_2_ group showed a significant decrease in the lung W/D value compared with resuscitation group, which indicates that H_2_ inhalation was benefit to relieve edema [6]. HRS administration also decreased pulmonary W/D weight ratio [11]. H_2_ inhalation alone decreased W/D ratio as well [5, 12].

Examination of cell counts and protein concentration in BALF is a particular technique to evaluate lung effusion and its character. Animals studies mentioned that CLP or LPS increased the cell counts and protein in BALF, which could be remitted with H_2_ inhalation [5, 10]. Xie et al. proved that H_2_ inhalation and HRS injection were both effective to reduce the cell counts, PMNs (Polymorphonuclears), and total protein increased by LPS [12].

Antioxidant enzymatic activity in lung (SOD and CAT) was suppressed and level of oxidative products (MDA and 8-iso-PGF2*α*) was increased in sepsis. H_2_ inhalation and HRS injection both could restrain oxidative stress [5, 6, 10, 11]. Some study presented that increased MPO in the lung of septic mice could be lightened by molecular hydrogen therapy [5, 6, 10, 12] but others presented that HRS injection had no effect of decreasing MPO level [11].

Inflammatory cytokines (TNF-*α*, HMGB1, IL-1*β*, IL-6, and IL-8) were increased while anti-inflammatory cytokines (IL-10) were decreased, in serum and lung in sepsis patients. Molecular hydrogen therapy could reduce the level of inflammatory cytokines [5, 6, 10] and increase the level of anti-inflammatory cytokines [10] in septic mice. There are also some researchers who considered molecular hydrogen therapy had no significant effect on the level of TNF-*α* and IL-10 [11, 12].

Liu et al. [16] combined H_2_ therapy with NO therapy in LPS-challenged mice and found that the combination therapy had significant interaction between the two and had more beneficial effect than H_2_ inhalation alone.

Generally speaking, lung structure was damaged by sepsis. Lung W/D ratios, cell counts and protein concentration in BALF, level of oxidative products, and inflammatory cytokines were found increased, while antioxidative enzyme activity and anti-inflammatory cytokines were found decreased. Although there still were controversies [11, 12], most researchers regarded molecular hydrogen therapy as a valid technique to alleviate all those pathologic changes.

### 5.3. Liver

Liver is one of the most important organs, but also one of the first organs to be affected during sepsis. Except for degree of oxidative stress reaction and inflammatory reaction, ALT and AST (aspartate aminotransferase) can also reveal hepatic function.

Histopathological changes in liver were shown in sepsis. Animal study shows liver histologic scores significantly increased in CLP group; O_2_ inhalation group and H_2_ inhalation group both showed much lower scores, which even had no difference to sham group [5, 10].

In addition, CLP mice developed significant liver injury, which was assessed by ALT and AST increase. H_2_ inhalation and HRS injection both could attenuate these abnormal changes [5, 10, 11]. Especially in Xie et al.'s study, H_2_ inhalation group even had no significant difference with sham group, indicating the dramatic effect of H_2_ therapy [5].

Oxidative stress reaction and inflammatory reaction of liver were similar with lung. Inflammatory cytokines like TNF-*α* and HMGB1 were increased, while anti-inflammatory cytokines like IL-10 were decreased in sepsis. Antioxidant enzyme activities (SOD and CAT) were decreased and oxidative products (8-iso-PGF2*α*) were increased. H_2_ inhalation alleviated those changes [5, 10].

Studies about liver damage and hepatic function in sepsis with molecular hydrogen therapy were in a small number. Even so, these results revealed a dramatic effect of molecular hydrogen therapy. It may indicate that molecular hydrogen therapy is much more efficacious in liver protection. More research is needed in this area.

### 5.4. Kidney

Acute kidney injury (AKI) is a common disease in septic patients and can aggravate the condition of septic shock patients, resulting in higher mortality. Except for degree of oxidative stress reaction and inflammatory reaction, blood urea nitrogen (BUN) and creatinine (Cr) can also reveal hepatic function.

H&E staining of kidney tissues exhibited edema in renal tubular epithelial, damaged brush border, and interstitial edema with hemorrhage in septic mice. Tubular epithelial cell damage was ameliorated in H_2_ inhalation group. The similar result showed in transmission electron microscopic analysis of glomerular filtration membrane [13]. Kidney histologic scores increased significantly in CLP group; it is marvelously alleviated in H_2_ inhalation group which even had no difference with sham group [5, 10].

Serum BUN and Cr were much higher in LPS or CLP group than in sham group. H_2_ inhalation group had significant reductions of serum BUN and Cr [5, 10, 11, 13]. But one study revealed there was no significant difference of BUN/Cr ratio in all the groups. As BUN/Cr ratio is used to analyze whether prerenal azotemia or tubular ischemia exists in AKI, molecular hydrogen therapy may not be as effective as we thought [13].

Oxidative stress reaction and inflammatory reaction of kidney were similar with lung and liver. Inflammatory cytokines (TNF-*α*, IL-6, and HMGB1) were increased, while anti-inflammatory cytokines (IL-10) were decreased in sepsis. Antioxidant enzyme activities (SOD and CAT) were decreased and oxidative products (MDA and 8-iso-PGF2*α*) were increased. H_2_ inhalation alleviated those changes [5, 10, 13]. However, in study of Liu et al., level of IL-10 had no change between all groups [13].

In spite of some dispute, molecular hydrogen therapy was considered as a useful method to alleviate structure damage of kidney, protect renal function, and resist inflammatory reaction and oxidative reaction.

### 5.5. Intestine

Sepsis leads to significant decrease in blood flow of the gastrointestinal tract. Hyperperfusion induces severe ischemia, hypoxia, and reperfusion injury. Researchers also work at molecular hydrogen therapy alleviating septic damage in intestine.

In animal study, after LPS manipulating, the structure of the small intestinal mucosa was damaged. Glands of the small intestine were destroyed. Edema of mucosal villi, neutrophil infiltration, and even intestinal ulceration was also commonly observed in sepsis. Resuscitation therapy worsened the damage mentioned above while H_2_ inhalation reduced the damage. The histologic score of LPS group was significantly higher than sham group, but the score of H_2_ inhalation group was significantly decreased compared with LPS group [6].

The serum diamine oxidase (DAO) activity reflects degree of intestinal mucosa epithelium cell impaired. Levels of DAO in sham group, LPS group, and H_2_ group were 4.32 ± 0.33 kU/L, 6.54 ± 0.68 kU/L, and 5.14 kU/L (*p* < 0.05), respectively [6]. The result demonstrated that H_2_ inhalation could protect epithelium cell of intestine from septic damage.

Oxidative stress reaction and inflammatory reaction of intestine were similar with lung, liver, and kidney. Inflammatory cytokines (TNF-*α*, IL-6, IL-8) were increased in LPS group. Antioxidant enzyme activity (SOD) was decreased and oxidative products (MDA) were increased in sepsis. H_2_ inhalation alleviated those changes.

The effect of molecular hydrogen therapy of intestinal damage in sepsis curing with molecular hydrogen therapy needs more research. According to the only literature of study, molecular hydrogen therapy protected intestine from sepsis.

## 6. The Impact of Molecular Hydrogen on Outcomes

All the research indicated molecular hydrogen therapy can improve survival rate of septic animal whatever the method of drug administration and sepsis inducing.

Zhang et al. compared 3 different ways to induce sepsis and the survival rate, respectively. The survival rates of LPS-induced septic mice at 24, 48, and 72 hr were 88.89%, 66.67%, and 66.67%. With HRS treatment, the survival rate increased to 100% (*p* < 0.05), 75%, and 75%, respectively. When challenged by feces injection, survival rates of mice at 24, 48, and 72 hr were 100%, 75%, and 75%. With HRS treatment, the survival rate increased to 85.71%, 85.71% (*p* < 0.05), and 85.71% (*p* < 0.05), respectively. Survival rates of CLP-induced septic mice at 24, 48, and 72 hr were 76.47%, 47.06%, and 35.23%. With HRS treatment, the survival rate increased to 72.73%, 72.73% (*p* < 0.01), and 54.54% (*p* < 0.01), respectively. The 3-day mortality rates after modeling were 33.33% (LPS model), 25% (feces model), and 64.7% (CLP model), while HRS treatment reduced them to 25%, 14.29% (*p* < 0.05), and 45.45% (*p* < 0.01), respectively [4]. There was still no denied data yet.

## 7. Safety Concerns

As mentioned before, there are 3 methods of molecular hydrogen therapy: inhalation of hydrogen (H_2_), oral intake of hydrogen-rich water, and injection of hydrogen-saturated saline. In low concentration (4.1% in pure oxygen or 4.6% in the air), hydrogen is neither explosive nor dangerous. Others thought it is safer to dissolve hydrogen into water and administrate the HRS by oral or by injection. However, the best way of molecular hydrogen therapy, the appropriated dosage, and the safety concerns are still to be discussed.

## 8. Conclusion

Molecular hydrogen therapy has a protective effect on sepsis, which has been proved by pathological biopsy, level of inflammatory factors/anti-inflammatory factors, oxidative stress reaction, behavioral experiment, and other related indicators of organ function. Although there is a dispute of affections of molecular hydrogen therapy in liver and kidney, the mainstream view shows molecular hydrogen therapy is benefit to organs, such as brain, lung, liver, kidney, and small intestine.

Molecular hydrogen therapy combining with oxygen therapy or fluid resuscitation can reduce oxygen free radical damage, the amount of fluid and vasoactive drugs, and the overload of liquid. As a result, molecular hydrogen therapy may reduce the complications of oxygen therapy and fluid resuscitation.

However, most of the study conclusion came from animal experiment while reports of clinical research were rare. Much more clinical evidence is still demanded.

In conclusion, molecular hydrogen therapy is a promising method to alleviate organ damage, improve outcome, and reduce mortality rate in sepsis.

## Figures and Tables

**Table 1:** The impact of changes of the biochemistry indicator level.

	Brain	Lung	Liver	Kidney	Intestine
Capase-3	HRS ↓	HRS ↓	/	/	/
SOD	HRS ↑,H_2_ ↑	HRS ↑	H_2_ ↑	H_2_ ↑	H_2_ ↑
ROS	H_2_ ↓, HRS ↓	/	/	/	/
MPA	H_2_ ↓, HRS ↓	HRS ↓	/	H_2_ ↓	H_2_ ↓
CAT	HRS ↑, H_2_ ↑	HRS ↑	H_2_ ↑	H_2_ ↑	/
8-iso-PGF2*α*	H_2_ ↓	HRS ↓	H_2_ ↓	H_2_ ↓	/
Nrf2	H_2_↑	/	/	/	/
TNF-*α*	H_2_ ↓	HRS ↓ or →	H_2_ ↓	H_2_ ↓	H_2_ ↓
IL-1*β*	H_2_ ↓	HRS ↓	/	/	/
IL-6	/	HRS ↓	/	H_2_ ↓	/
IL-8	/	HRS ↓	/	H_2_ ↓	/
HMGB1	H_2_ ↓	HRS ↓	H_2_ ↓	/	H_2_ ↓
IL-10	H_2_ ↑	HRS ↑ or →	H_2_ ↑	H_2_ ↓ or →	/
MPO	/	HRS ↑ or →	/	/	/
Others	Cognitive impairment: HRS is less than sepsis group; HRS is similar to sham group	(1) W/D weight ratio: H_2_ ↓, HRS ↓ (2) BALF cell counts: H_2_ ↓	ALT, ACT: H_2_ ↓, HRS ↓ H_2_ group is similar to sham group	BUN, Cr: H_2_ ↓	DAO: H_2_ ↓
References	[7–9]	[5, 6, 10–12]	[5, 10, 11]	[5, 10, 11, 13]	[6]

HRS: hydrogen-rich water injection or oral-taken group.

H_2_: H_2_ inhalation group.

SOD: superoxide dismutase.

CAT: catalase.

HMGB1: high mobility group box 1.

IL: interleukin.

W/D: wet/dry.

BALF: bronchoalveolar lavage fluid.

ALT: alanine aminotransferase.
